# Comparative Transcriptomic Analysis Reveals the Effects of Drought on the Biosynthesis of Methyleugenol in *Asarum sieboldii* Miq.

**DOI:** 10.3390/biom11081233

**Published:** 2021-08-18

**Authors:** Fawang Liu, Tahir Ali, Zhong Liu

**Affiliations:** School of Pharmacy, Shanghai Jiao Tong University, Shanghai 200240, China; fawang90@126.com (F.L.); Tahirali@sjtu.edu.cn (T.A.)

**Keywords:** *Asarum sieboldii* Miq., drought, RNA-seq, volatile oil, methyleugenol

## Abstract

*Asarum sieboldii* Miq., a perennial herb in the family *Aristolochiaceae*, is widely used to treat colds, fever, headache and toothache in China. However, little is known about the drought-tolerance characteristics of *A. sieboldii*. In this study, to elucidate the molecular–genetic mechanisms of drought-stress tolerance of *A. sieboldii*, RNA-seq was conducted. In total, 53,344 unigenes were assembled, and 28,715 unigenes were annotated. A total of 6444 differential-expression unigenes (DEGs) were found, which were mainly enriched in phenylpropanoid, starch and sucrose metabolic pathways. Drought stress revealed significant up-regulation of the unigenes encoding PAL, C4H, HCT, C3H, CCR and IGS in the methyleugenol-biosynthesis pathway. Under the condition of maintaining drought for 15 days and 30 days, drought stress reduced the biosynthesis of volatile oil by 24% and 38%, respectively, while the production of key medicinal ingredients (such as methyl eugenol) was increased. These results provide valuable information about the diverse mechanisms of drought resistance in the *A. sieboldii*, and the changes in the expression of the genes involved in methyleugenol biosynthesis in response to drought stress.

## 1. Introduction

Climate change has a significant effect on agriculture. Among the various environmental stresses, drought is the most common and the most severe abiotic stress, as it has a profound impact on crop productivity and survival [1]. Drought stress has a significant effect on photosynthesis and antioxidant metabolism, limiting photosynthesis through stomatal closure, reducing gas exchange and increasing active oxygen, leading to a decrease in plant growth and productivity [2]. During periods of drought, plants have developed comprehensive mechanisms, such as metabolic alteration, signal transduction, and differential gene expression, to maintain balance, optimizing yield and survival. In general, three types of resistance strategies have evolved to respond to drought environments: stress escape, stress avoidance and stress tolerance [3,4]. Many studies have provided valuable insights into the molecular and cellular mechanisms by which plants responds to drought in recent years. However, these regulatory mechanisms, which coordinate between drought-stress tolerance and plant growth, are still not fully understood [3,5,6,7].

*Asarum sieboldii*, a traditional Chinese medicinal herb, is mainly distributed in central and eastern China continent, and Japan [8]. Its medicinal use was first recorded in the Shennong Compendium of Materia Medica (Shennong Bencaojing) in about A.D. 25–A.D. 220 [9]. Its essential oil has anti-inflammatory, antibacterial and anti-allergenic properties; it also exhibits acaricidal activity against *Dermatophagoides farinae* [10]. Today, *A. sieboldii* is used to treat colds, fever, aphthous stomatitis, toothache, gingivitis and rheumatoid arthritis in clinics and hospitals [11,12,13,14]. Methyleugenol (4-allyl-1,2-dimethoxybenzene), a phenylpropene compound, is widely distributed in many aromatic plants, such as *Acacia senega l*(L.) Willd. (Mimosaceae), *Cinnamomum verum* J.S. Presl. (Lauraceae), *Gentiana lutea* L. (Gentianaceae) and *Rosmarinus officinalis* L. (Lamiaceae). Besides its medicinal uses, it is also widely used in foodstuffs as a flavoring agent [15]. Methyleugenol is considered as the key constituents of *Asarum*’s volatile oil [16]. The biosynthetic pathway of methyleugenol in *A. sieboldii* and basil (*Ocimum basilicum* L.) has been proposed, and several enzymes (PAL, C4H, 4CL, CCR, CAD, EGS, EOMT) were identified, playing a pivotal role in the regulation of methyleugenol biosynthesis [16,17,18]. It has been reported that drought stress reduces the expression level of 4CL and C4H, but increases the expression of EOMT, and therefore increases methyleugenol content in the essential oil of basil [17] and Iranian basil [18]. However, so far, the expression levels of these genes, involved in the biosynthesis of methyleugenol in response to drought stress in *A. sieboldii*, have not been fully explored.

The next-generation sequence (NGS) technique, known as RNA sequence (RNA-Seq), is a cost-efficient tool for transcriptome studies [19]. RNA-Seq has been successfully used in many plants such as maize [20], creeping bentgrass [21], mulberry [22], and *Artemisia sphaerocephala* [23]. RNA-Seq is used in biological studies worldwide to understand biological processes better, and it has primarily been applied to studying gene-expression responses to various stresses. However, there is a lack of published reports using RNA-Seq to analyze the regulation of gene expression of *A. sieboldii* under drought stress.

Our previous works used transcriptomic analysis to mine many key genes involved in methyleugenol biosynthesis in *A. sieboldii* [16,24]. In this study, we further used the Illumina sequence to perform a deep transcriptome sequence of *A. sieboldii* to compare between genome-wide differential expressions under a drought treatment and normal conditions. The RNA sequence data were de novo assembled and annotated. Moreover, differentially expressed genes were characterized, and potential candidate genes in the methyleugenol-biosynthesis pathway were identified. Our study provides useful information and the first comprehensive discovery of the *A. sieboldii* transcriptome under drought stress conditions. Furthermore this information provides a starting point for elucidating the molecular mechanisms involved in defining the response of *A. sieboldii* against drought stress, and improving methyleugenol content using genetic engineering.

## 2. Materials and Methods

### 2.1. Plant Materials and Drought-Stress Procedures

Two-year-old plants of *A. sieboldii* were transplanted from Ningqiang county, Hanzhong, Shanxi Province, China, in 2019 and identified by Dr. Z. Liu [16]. Plants were grown in plastic pots of soil containing vermiculite and perlite. Plant’s growth conditions were controlled, and as follows: room temperature was 23 ± 2 °C, photoperiod was 18 h light and 6 h dark and illumination strength was 200 μmol/(m^2^·s). Plants at the early flowering stage were used for drought experiments in this study. For drought-stress treatment, we used plants that were irrigated once a week as a negative control, while irrigation, for the treatment plants, was stopped after 15 days or 30 days. For each group, three individual lines were grown in one pot. All plant tissue, including root, rhizome, petiole and leaf, were collected from each plant separately, then frozen immediately in liquid nitrogen. The tissue samples were stored at −80 °C before use.

### 2.2. Volatile-Oils Extraction and GC-MS Analysis

Volatile-oil extraction from *A. sieboldii* was experimentally determined using Chinese Pharmacopoeia 2015 Edition’s (General Rules 2204) [25] hydrodistillation method. Roots and rhizomes (100 g of wet weight) were cut into small pieces and transferred into a 500 mL reflux flask, which contained 250 mL water, followed by hydrodistillation for 5 h, using a Soxhlet extraction instrument. Extraction was stopped when oil was no longer released. The oil was collected and dried in anhydrous sodium sulphate.

GC-MS analysis was performed using an Agilent 7890B-7000D, equipped with an Agilent 122-5532UI column (30 m × 250 μm × 0.25 μm). Oven temperature conditions were 50 °C for 1 min, followed by programmed heating, from 50 °C to 100 °C, at a rate of 10 °C/min and from 100 °C to 160 °C at a rate of 2 °C/min, finally heating from 160 °C to 300 °C at a rate of 10 °C/min, held for 5 min. The carrier gas, helium, was adjusted to a linear velocity of 1 mL/min; the split ratio was 5:1 and the split flow was 5 mL/min. The ion source temperature was 250 °C and the ionization energy was 70 eV, with a mass scan range of 33 AMU–500 AMU. Compounds were identified by matching their mass data with the NIST (National Institute of Standards and Technologies) mass-spectra library. GC-MS data were analyzed using the MSD Chemstation software (version F.01.03.2357).

### 2.3. Library Construction and Illumina Sequencing

The same tissues from three independent plants of one treatment were pooled together based on root, rhizome, petiole and leaf blade, accordingly. RNA samples were isolated using a TRIzol reagent following the manufacturer’s protocol (Invitrogen, Carlsbad, CA, USA). The RNA purity and concentration were checked using a NanoDrop ND-1000 spectrophotometer (Thermo Scientific, Waltham, MA, USA) and an Agilent 2100 Bioanalyzer (Agilent, Palo Alto, CA, USA).

For library preparation and sequencing, a 1 μg RNA per sample was used as input material for the RNA preparation. Sequencing libraries were generated using the NEBNext^®^Ultra™ RNA Library Prep Kit for Illumina (NEB, Ipswich, MA, USA) following provided instructions. The PCR products were purified using a Beckman Agencourt AMPure XP (Beckman Coulter, Brea, CA, USA) and the library quality was assessed on an Agilent Bioanalyzer 2100. The libraries’ preparation was conducted by Biomarker Biotechnology, Co., Ltd. (Beijing, China), and then sequenced using the Illumina Hiseq 2000 platform. A two-terminal sequencing method was used, finally generating paired-end reads with 150 bp in length.

### 2.4. Quality Control and De Novo Transcriptome Assembly

For quality control, raw reads of the fastq format were firstly processed through in-house Perl scripts (version 5.18.4). In this step, clean reads were obtained by removing reads containing adapter or containing ploy-N (>10%) and low-quality reads (Q30 > 85%) that did not meet the quality threshold. At the same time, the Q20, Q30, GC-content and sequence duplication levels of the clean reads were calculated. All downstream analyses were based on these clean and high-quality data.

To date, there is no report in the literature on the genome data of *A. sieboldii*. Therefore, it was necessary to splice the clean data. For transcriptome assembly, the resulting paired-end clean reads were clustered using TGICL software (http://www.tigr.org/tdb/tgi/software/ (accessed on 17 December 2019)) [26]. Paired-end sequences were separated into two files (left.fq file and right.fq file). Transcriptome assembly was accomplished based on the two files using Trinity software (v2.5.1) with its min_kmer_cov parameter set to two and all other parameters set to default [27]. The assembly results were further sequenced and de-redundantly processed using sequence clustering software (TGICL) to obtain long nonredundant unigene sequences.

### 2.5. Gene Functional Annotation

The resulting unigenes were annotated with BLASTx by comparison against the database of nonredundant protein sequences (NR) [28], Clusters of Orthologous Groups of proteins (COG) [29], euKaryotic Orthologous Groups (KOG) [30], SWISS-PROT [31], Kyoto Encyclopedia of Genes and Genomes (KEGG) [32], Gene Ontology (GO) [33], eggNOG [34] and Protein family (Pfam) [35]. Using HMMER software (v3.1b2), we compared our output with the Pfam database, then set the BLAST E-value parameter at less than 1 × 10^−5^ and the HMMER parameter E-value at less than 1 × 10^−10^ [35,36]. The aligned proteins with the highest similarity were used for functional annotation.

### 2.6. Differential Expression Analysis

In order to identify genes with different expression patterns between the drought treatment group and the normal group, differentially-expressed-genes (DEGs) analysis was performed. For each sample, the gene abundances were estimated by expectation-maximization (RSEM) [37]. The fragments per kilobase of exon model per million mapped reads (FPKM) was used to represent the expression abundance of the corresponding differentially expressed genes, because the FPKM method can eliminate the influence of different gene lengths and sequencing discrepancies on the gene-expression calculation [38,39,40]. Based on the length of the gene and the number of reads mapped to the gene, the FPKM value of each gene was calculated.

Differential expression analysis was performed using the EBSeq package (v1.6.0) [41]. The resulting *p* values were adjusted using Benjamini and Hochberg’s approach to control false discovery rates [42]. A false discovery rate (FDR) of <0.01 and a difference in fold change (FC) of ≥2 were set as screening criteria for significant differential expression between two samples. The identified DEGs were further analyzed using the GO and KEGG databases. GO enrichment analyses were implemented with the topGO R packages (v2.28.0) based on the Kolmogorov–Smirnov test, to search for and map all significantly enriched GO terms. KEGG enrichment was conducted using KOBAS software (v2.0) [43]. Gene expression-trend analyses of DEGs were performed using the cluster package (https://cran.r-project.org/web/packages/cluster/index.html (accessed on 17 December 2019).

### 2.7. Real-Time qPCR Validation of RNA-Seq Data

The expression levels of six DEGs in the phenylpropanoid biosynthesis pathway, which encoded PAL, HCT, CCOMT, CCR, CAD and IGS, were verified by qRT-PCR. Total RNA was extracted from the rhizome in control- and drought-treated plants. First-strand cDNA was synthesized from about 1 μg of total RNA using FastQuant RT Kit (TIANGEN, China). The qRT-PCR assay was conducted on an Applied Biosystems StepOnePlus™ systems (Bio-Rad, Hercules, CA, USA), using 2 × ChamQ Universal SYBR qPCR master mix (Vazyme, China). The relative expression was normalized to the expression level of the internal control 18S rRNA and calculated by the 2^−ΔΔCt^ method (Supplementary Material Table S1) [44,45].

## 3. Results

### 3.1. Volatile Oils Content and Composition

**Figure 1:**
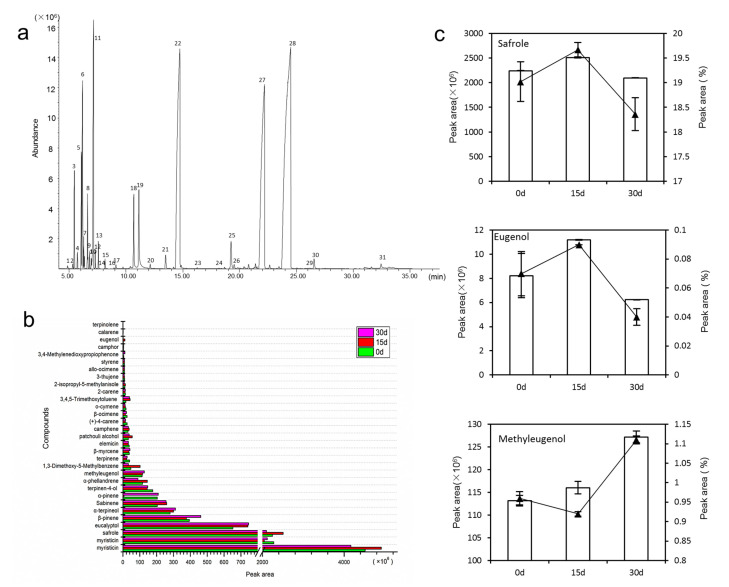
The changes of volatile oil content and its composition of *A. sieboldii* under drought stress. (**a**) the 31 components identified in volatile oil by GC-MS; (**b**) the content change in 31 components under drought and control conditions; (**c**) the relative content of safrole, eugenol and methyleugenol in drought and control.

The volatile oil in the root and rhizome of *A. sieboldii* was extracted and dried in anhydrous sodium sulphate. The content of oil was 0.34 g (0.34%), 0.26 g (0.26%), and 0.21 g (0.21%) in 0 days, 15 days and 30 days of drought, respectively. The mass spectrometry data were compared with the NIST14 database, and referenced against the data reported in the literature. A total of 31 components were identified (Figure 1a,b, Table S2). The most abundant components in volatile oil were myristicin (19.31~38.24%), safrole (19.02%), eucalyptol (5.53%), α-pinene and β-pinene (α/β-pinene, 1.73%, 3.34%), α-terpineol (2.37%), sabinene (1.73%), terpinene-4-ol (1.49%), α-phellandrene (0.99%) and methyleugenol (0.96%). In the drought treatment conditions, dynamic changes in safrole, eugenol and methyleugenol were detected (Figure 1c). Peak-area and relative-peak-area percentage were used for relative quantification. Our results showed that, under continuous drought, the contents of safrole and eugenol increased at moderate drought and then decreased. However, the content of methyleugenol showed a continuously increasing trend.

### 3.2. Sequencing Output and Assembly

RNA quality was determined, and the RNA integrity number (RIN) was calculated. The RNA samples were 1.2–6.9 μg, with OD_260_/OD_280_ = 2.01–2.17 and RIN = 7.7–9.7; the quality was satisfactory for constructing cDNA libraries. cDNA libraries were sequenced using the Illumina HiSeq™ 2000 platform. After deletion of adaptor-polluted, redundant and other low-quality sequences, a total of 77.38 Gb clean reads were obtained, and each sample had more than 6.14 Gb clean reads. The Q30 scores (sequencing error rate, 0.1%) were more than 94%, and GC contents were more than 45%. After filtration, the Trinity tool was used to assemble independent, high-quality, clean sequences in each library, which were further merged, generating 228,618 transcripts and 53,344 unigenes. The transcripts and unigenes were 38,789,699 bp length and 51,209,482 bp in length, respectively. The mean sizes of total transcripts and unigenes with N50s were 2423 bp and 1469 bp. The mean lengths of total transcripts and unigenes were 1696.68 bp and 959.99 bp, respectively. Among the 53,344 unigenes, 23,455 (43.97%) unigenes were between 300 bp and 500 bp; 15,156 (28.41%) unigenes were between 500 bp and 1000 bp; 8559 (16.04%) unigenes were between 1 kb and 2 kb; 6174 (11.57%) unigenes were more than 2 kb (Table 1). The quality of unigenes was sufficient to perform further analysis.

### 3.3. Functional Annotation and Classification

**Figure 2:**
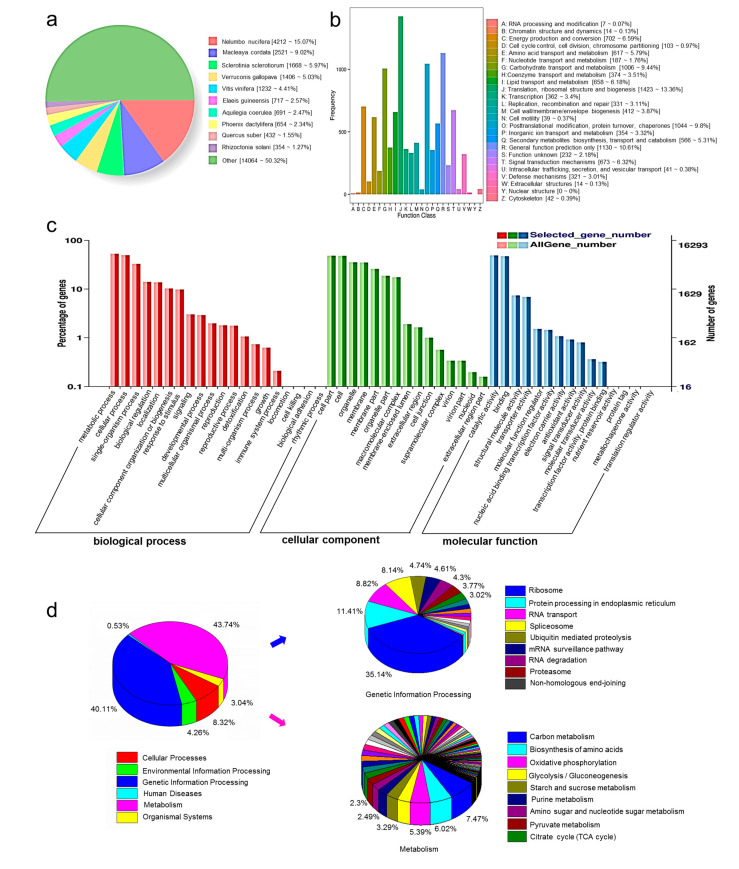
NR annotated species distribution and functional annotation of unigenes. (**a**) BLAST top hit species distribution, (**b**) COG classification, (**c**) GO classification and (**d**) KEGG pathways classification.

In total, 28,715 unigenes were annotated. Among these annotated unigenes, 16,313 unigenes were less than 1000 bp, 12,402 unigenes were more than 1000 bp. A total of 27,995 unigenes were annotated with the NR database, followed by the eggNOG database with 26,155 unigenes annotated (Table 2). BLASTx identified that half of the 27,995 unigenes shared high similarity with ten plant species in the NR database. Among them, 4212 unigenes had variable numbers of hits with *Nelumbo nucifera* (15.07%), followed by *Macleaya cordata* (2521, 9.02%), *sclerotinia sclerotiorum* (1668, 5.97%), *verruconis gallopava* (1406, 5.03%) and *vitis vinifera* (1232, 4.41%) (Figure 2a).

COG annotation aligned 9620 unigenes with functional classification; among them, 1423 (13.36%) unigenes were related to the translation, ribosomal structure and biogenesis, 1044 (9.8%) unigenes were related to posttranslational modification, 1006 (9.44%) unigenes were related to carbohydrate transport and metabolism, 673 (6.32%) unigenes were related to signal transduction mechanisms, 566 (5.31%) unigenes were related to secondary metabolites biosynthesis, transport and catabolism, etc. (Figure 2b).

The 16,293 unigenes annotated with GO were assigned to three categories, biological processes (BP), cellular components (CC) and molecular functions (MF). In BP, unigenes were enriched in metabolic processes (8639, 53.02%). In CC, unigenes were mostly distributed in cells (7836, 48.09%) and cell parts (7861, 48.25%). In MF, unigenes were mainly distributed in catalytic activity (7991, 49.05%) and binding (7673, 47.09%) (Figure 2c).

Assembled unigenes were assigned to different metabolic pathways in the KEGG database. Among the 10,789 unique mapped sequences, 3059 (43.74%) unigenes were assigned to metabolism, 2805 (40.11%) unigenes were assigned to genetic information processing, 582 (8.32%) unigenes were assigned to cellular processes and others, including environmental information processing (298 unigenes), organismal systems (213 unigenes) and human diseases (37 unigenes). With respect to metabolism, the top five most-annotated pathways were carbon metabolism (497 unigenes), biosynthesis of amino acids (401 unigenes), oxidative phosphorylation (359 unigenes), glycolysis/gluconeogenesis (235 unigenes) and starch and sucrose metabolism (219 unigenes). In the genetic information processing, the top five annotated pathways were ribosome (1127 unigenes), protein processing in the endoplasmic reticulum (366 unigenes), RNA transport (283 unigenes) and spliceosome (261 unigenes) (Figure 2d).

### 3.4. Differentially Expressed Genes in Root and Rhizome

**Figure 3:**
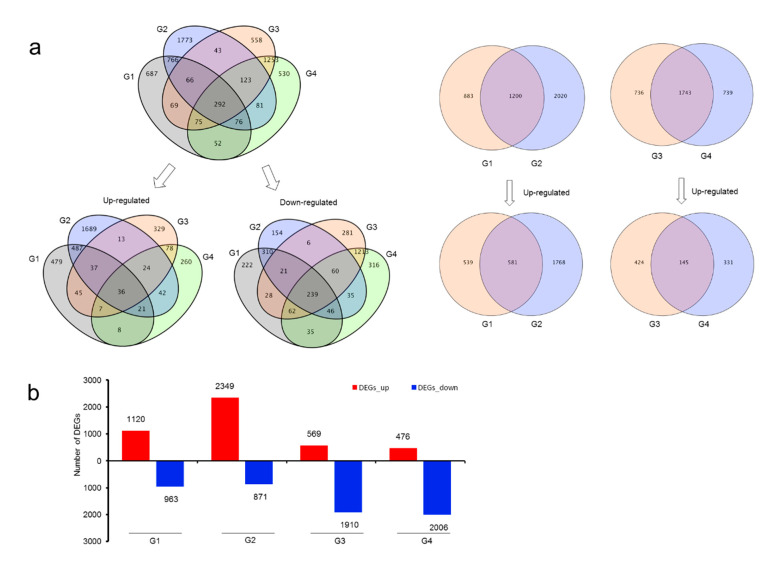
Differentially expressed genes in root and rhizome. (**a**) Venn diagram of DEGs in root and rhizome between drought and control group. (**b**) Up-regulated and down-regulated DEGs in root and rhizome. Abbreviation G1 (R h-0_vs._Rh-15), G2 (Rh-0_vs._Rh-30), G3 (Ro-0_vs._Ro-15), G4 (Ro-0_vs._Ro-30).

Total of 6444 unigenes of differential expression (with FDR < 0.01 and FC ≥ 2) were found in root and rhizome in drought treatment compared with normal growing ones (Table S3). Among these DEGs, 292 DEGs were shared both in root and rhizome; 3555 unigenes were found only up-regulated, while 3028 unigenes were found only down-regulated (Figure 3a).

In rhizome, a total of 4103 DEGs were found, 1200 DEGs were shared in both Rh-0_vs._Rh-15 (G1) and Rh-0_vs._Rh-30 (G2); among these shared genes, 581 unigenes were up-regulated. In the root, a total of 3218 DEGs were found, among them, 1743 DEGs were shared in both Ro-0_vs._Ro-15 (G3) and Ro-0_vs._Ro-30 (G4), and 145 of these shared genes were up-regulated.

In rhizomes, the up-regulated DEGs in drought for 30 days were more than drought for 15 days. However, in roots, the amount of DEGs did not change too much. It indicated that the rhizome senses the environment to adjust quickly in abiotic stress than other plant tissues. Compared with the roots, the rhizomes have a higher degree of lignification, and their response to water loss is obvious. Therefore, the amount of DEGs gradually increased with drought duration, and the number of up-regulated DEGs increased from 1120 to 2349 (Figure 3b).

### 3.5. GO and KEGG Enrichment of DEGs

**Figure 4:**
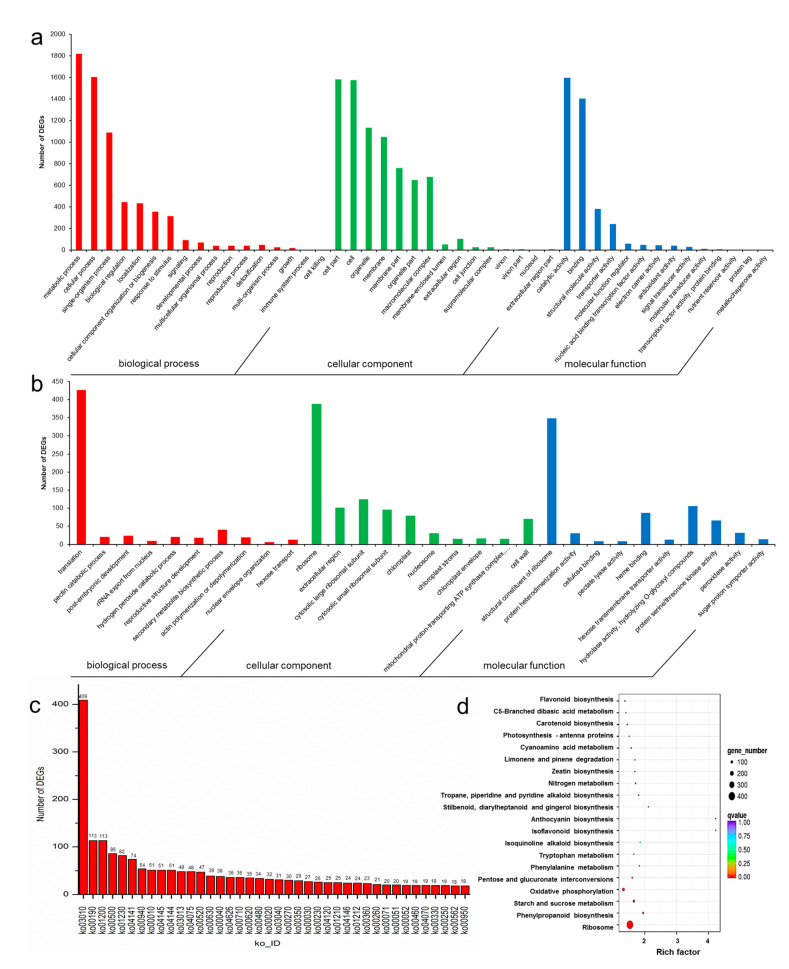
GO and KEGG analysis of DEGs in root and rhizome. (**a**) GO classification of DEGs in root and rhizome, (**b**) GO enrichment of DEGs in root and rhizome, (**c**,**d**) KEGG classification and KEGG enrichment of DEGs in root and rhizome.

The 6444 DEGs in roots and rhizome were annotated into 35 GO categories. In BP, the categories with the largest number of DEGs were the metabolic process (1819 unigenes), cellular process (1603 unigenes) and single-organism process (1090 unigenes). In CC, most of the DEGs were annotated to cell part (1580 unigenes), cell (1574 unigenes), organelle (1133 unigenes) and membrane (1048 unigenes). In MF, DEGs were mainly annotated to catalytic activity (1594 unigenes) and binding (1404 unigenes) (Figure 4a). For the GO enrichment of DEGs, the top 10 categories in BP, CC and MF were selected for analysis. GO enrichment of BP mainly focused on translation (1457 unigenes); in CC, it was focused primarily on the ribosome (1156 unigenes), and in MF, it was focused on the structural constituent of ribosome (1059 unigenes) (Figure 4b, Table S4).

KEGG annotation showed that these 6444 DEGs were annotated into 124 pathways, and the pathway with the most significant number of DEGs was ribosome (ko03010), followed by oxidative phosphorylation (ko00190) and carbon metabolism (ko01200), which had 113 DEGs each; the top 40 pathways with the most annotated genes were used for analysis. The KEGG enrichment analysis of DEGs was done with *p* < 0.05 and the top 20 enriched pathways were listed, which were ribosome (409 unigenes), phenylpropanoid biosynthesis (54 unigenes), starch and sucrose metabolism (86 unigenes), oxidative phosphorylation (113 unigenes), pentose and glucuronate interconversions (38 unigenes), phenylalanine metabolism (23 unigenes), tyrosine metabolism (29 unigenes) and isoquinoline alkaloid biosynthesis (18 unigenes) (Figure 4c,d, Table S5).

### 3.6. DEGs Related to Methyleugenol Biosynthesis

**Figure 5:**
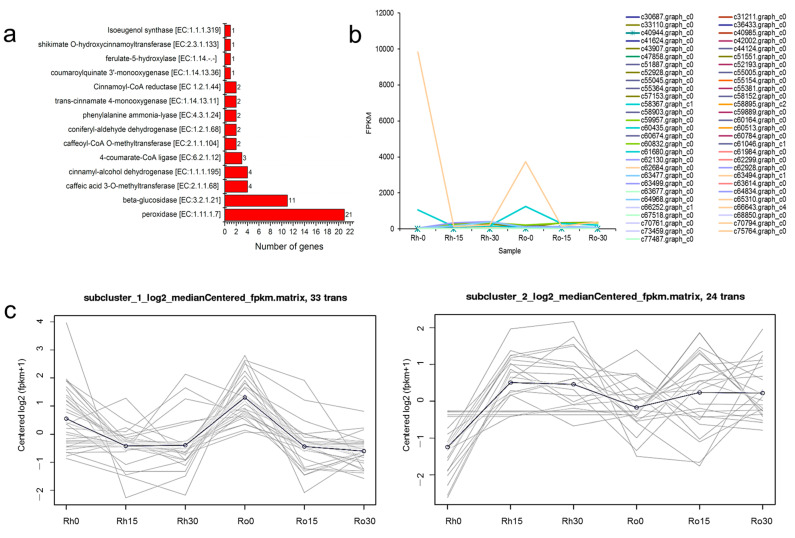
Expression profiles of 57 DEGs related to phenylpropanoid biosynthesis. (**a**) Number of DEGs in the phenylpropanoid biosynthesis pathway, (**b**) FPKM value of 57 DEGs, (**c**) clusters analysis of 57 DEGs.

**Figure 6:**
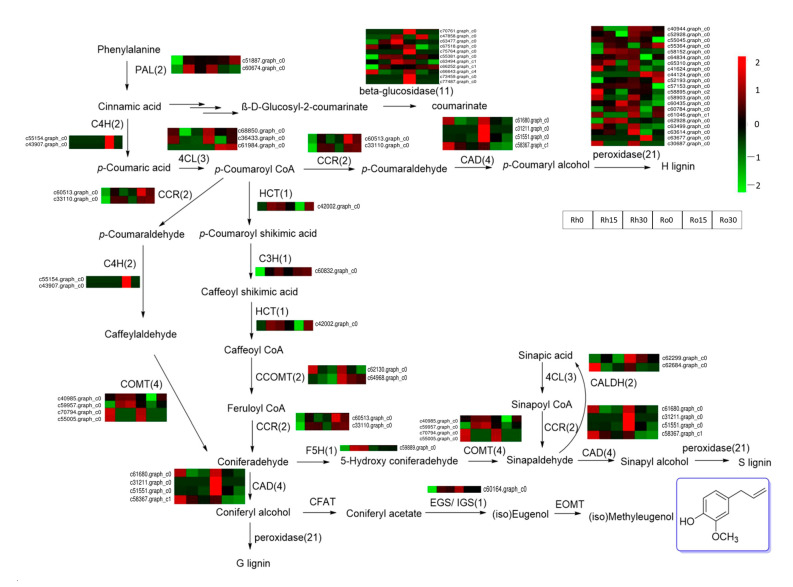
Changes in the expression level of the methyleugenol biosynthesis related genes. 57 DEGs were used for the heatmap, and the color indicates the FPKM value (log10) of the unigenes encoding the corresponding enzyme. The six colored boxes from left to right represent the gene expression value in Rh-0, Rh-15, Rh-30, Ro-0, Ro-15, and Ro-30. Numbers of putative unigenes were given in parentheses.

A total of 57 DEGs in the phenylpropanoid biosynthesis pathway were identified (Figure 5 and Figure 6, Table S6). These DEGs encode 14 enzyme families, including 21 peroxidase, 11 beta-glucosidase, 4 caffeic acid 3-O-methyltransferase (COMT), 4 cinnamyl-alcohol dehydrogenase (CAD), 3 4-coumarate-CoA ligase (4CL), 2 caffeoyl-CoA O-methyltransferase (CCOMT), 2 coniferyl-aldehyde dehydrogenase (CALDH), 2 phenylalanine ammonia-lyase (PAL), 2 trans-cinnamate 4-monooxygenase (C4H), 2 Cinnamoyl-CoA reductase (CCR), 1 coumaroylquinate 3′-monooxygenase (C3H), 1 ferulate-5-hydroxylase (F5H), 1 shikimate O-hydroxycinnamoyltransferase (HCT) and 1 isoeugenol synthase (IGS) (Figure 5a). Gene expression-level analysis found that two genes (c66643.graph_c4 and c61680.graph_c0) showed a higher FPKM value in rhizome and root. They were annotated to beta-glucosidase and cinnamyl-alcohol dehydrogenase (Figure 5b). Despite the phenylpropanoid biosynthesis pathway, some of these encoding genes were also involved in phenylalanine (ko00360), cyanoamino acid (ko00460), starch and sucrose (ko00500), flavonoid (ko00941), stilbenoid, diarylheptanoid and gingerol (ko00945), ubiquinone and other terpenoid-quinone (ko00130) pathways. Gene-co-expression trends of these 57 DEGs were analyzed and their DEGs were clustered into 2 profiles; as cluster 1 had 33 DEGs, and cluster 2 had 24 DEGs, it is obvious that these genes were down-regulated in cluster 1 and up-regulated in cluster 2 (Figure 5c).

Under drought for 15 days, 17 genes were found up-regulated in the rhizome, including three genes encoding beta-glucosidase, two genes encoding COMT, one gene encoding C3H, one gene encoding F5H, five genes encoding peroxidase, two genes encoding PAL, one gene encoding IGS and one gene encoding HCT, among these genes, one CCR encoding gene c33110.graph_c0 and one IGS encoding gene c60164.graph_c0 kept upregulation in drought. In roots, several genes were up-regulated, including genes encoding 4CL (1), beta-glucosidase (2), COMT (1), peroxidase (4), CCR (1) and HCT (1). We also found two genes, encoding C4H, to be up-regulated. However, these two genes were not detected in rhizomes. All these 57 DEGs, annotated to phenylpropanoid biosynthesis, were involved in the biosynthesis of many natural compounds, such as flavonoids, coumarins, furanocoumarins, catechins and G/H/S type lignin; also, these enzymes were upstream of the biosynthesis of methyleugenol. Methyleugenol was the major pharmaceutical component in *A. sieboldii*, and the biosynthesis pathway of methyleugenol, derived from the phenylpropanoid biosynthesis pathway. These 57 DEGs should supply valuable information for understanding the methyleugenol biosynthesis mechanism in a drought-stress environment. However, we have not found any DEGs annotated to EGS and EOMT in our transcriptome database. Interestingly, a gene encoding IGS was found and upregulated in the rhizome, which may accumulate the (iso) eugenol content and leading to an increase in methyleugenol content under drought stress.

### 3.7. qRT-PCR Validation

**Figure 7:**
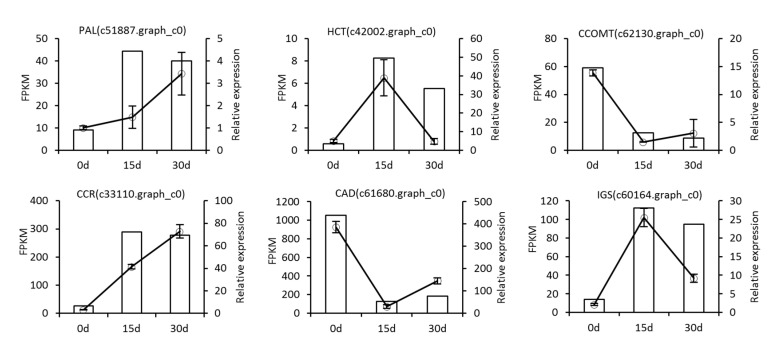
qRT-PCR validation of the expression of six DEGs involved in methyleugenol biosynthesis pathway. The x-axis shows the three samples, the y-axis shows the relative quantitative expression level and FPKM value of each unigene.

In this study, to validate the reliability of Illumina sequencing, six DEGs related to methyleugenol biosynthesis at different stages of drought were evaluated using qRT-PCR. The results showed a significant consistency between the qRT-PCR and transcriptome analyses (Figure 7), which indicated that the sequencing results were accurate, and the transcriptome data was reliable for genes analysis. For example, the expression levels of CCR (c33110.graph_c0) were much higher in drought groups (15 and 30 day’s drought) than in the control. However, differences in several individual genes were existed, such as HCT (c42002.graph_c0) and IGS (c60164.graph_c0). The little differences between the results of qRT-PCR and RNA-seq may be due to that some primers used in qRT-PCR were not optimal for amplification the target genes.

## 4. Discussion

### 4.1. Drought Stress Reduce Volatile Oil Content

Roots play a crucial role in the growth and development of a plant. The root system first senses a decrease in soil water, triggering a series of stress-responsive mechanisms to hold water [46]. In a drought environment, the elongation speed of the root tip slows down, and gradually starts depositing suberin, which leads to plant injury or even death [47]. According to the Chinese Pharmacopoeia, the most effective component of *A. sieboldii* is volatile oil, which mainly exists in the underground parts of the plant, namely the roots and rhizome [9]. Our results showed that the volatile oil in roots and rhizome decreased by 24% and 38% in 15 days and 30 days of drought treatment, respectively. The change in volatile oil content showed that drought seriously inhibited the production of volatile oil. Compared with 15 days of drought, the decrease rate of volatile oil content slowed under 30 days’ drought, which indicated that the effect of early drought on plants was greater than that of later drought.

Methyleugenol is the active component in the volatile oil of *A. sieboldii*, while safrole is aa toxic component and is considered a weak hepatocarcinogen, genotoxic and a transplacental carcinogen [48,49,50]. GC-MS analysis showed that eugenol content increased significantly after 15 days of drought, which was 1.59 times higher than that of the control group. The content of safrole increased slightly, which was 1.04 times higher than the control group. After 30 days of drought, the content of eugenol and safrole decreased, which were 0.82 times and 0.97 times those of the control group. However, the content of methyleugenol kept increasing (from 0.94% to 1.1%). Drought stress increased the content of methyleugenol, which was also observed in Basil (*Ocimum basilicum* L.) [17].

Transcriptome data-mining revealed that there was no gene annotated as eugenol synthase (EGS). However, one gene, c60164.graph_c0, was annotated as isoeugenol synthase (IGS). The gene expression values (FPKM) were 13.74, 112.33, and 94.68 in the 0 days, 15 days and 30 days of drought. It kept upregulating in drought stress compared with the control. The expression level of the gene (c60164.graph_c0) was consistent with the change of eugenol content, i.e., the gene expression level kept increasing at early drought and then decreased. It indicates that drought affects the production of eugenol or isoeugenol by regulating the expression of eugenol synthase or isoeugenol synthase encoding gene.

### 4.2. Drought Stress Induced the Gene Differentially Expressed in Methyleugenol Biosynthesis Pathway

A total of 57 DEGs were annotated to the phenylpropanoid biosynthesis pathway (Table S6). Among these genes, 18 genes involved in the methyleugenol biosynthesis, encoding 9 enzymes (PAL, C4H, 4CL, HCT, C3H, CCOMT, CCR, CAD, IGS). PAL is the first enzyme in the methyleugenol biosynthesis pathway [17]. There were two DEGs, which were annotated as PAL (c60674.graph_c0 and c51887.graph_c0), these two genes were up-regulated in rhizome under drought stress. C4H catalyzes the hydroxylation of trans-cinnamic acid to 4-hydroxycinnamate (p-coumaric acid), which is the second enzyme in the methyleugenol biosynthetic pathway [51]. In the roots, two genes, encoding C4H, showed upregulation after a drought of 15 days. Moreover, one gene, c42002.graph_c0 encoding HCT, was also up-regulated under drought. It is worth noting that a gene (c33110.graph_c0), encoding CCR, had a higher expression level, and was also continuously up-regulated under drought. Isoeugenol synthetase (IGS) or eugenol synthetase (EGS) is a specific enzyme that catalyzes the synthesis of isoeugenol (eugenol). It is also the penultimate enzyme in the methyleugenol biosynthesis pathway of *A. sieboldii*. The change in EGS expression level can directly affect the product of methyleugenol. In the pathway, terpinyl acetate was transferred to eugenol (isoeugenol) under the catalysis by eugenol (isoeugenol) synthetase, and then generated methyleugenol or isomethyleugenol under the action of eugenol O-methyltransferase (EOMT). A gene (c60164.graph_c0) functionally annotated isoeugenol synthetase, which was continuously up-regulated in rhizomes under drought, may be responsible for the increase of methyleugenol in the volatile oil. Basil (*Ocimum basilicum L*.) is a medicinal plant, and methyleugenol is the most crucial constituent of basil essential oil. It is reported that drought stress down-regulated the expression levels of 4CL and C4H genes, and up regulated EOMT, improving the amount of methyleugenol in basil [17]. In our study, the content of methyleugenol was also increased under drought stress (increased by 17.02%). Our results demonstrated that the expression levels of HCT, CCR, and IGS are correlated with methyleugenol content. Therefore, drought stress possibly increases the methyleugenol level, by increasing the expression of HCT, CCR, and IGS.

The process from eugenol to methyleugenol was catalyzed by eugenol-O-methyltransferase (EOMT). The annotated unigene data were fully screened, but there was no gene functionally annotated as EOMT. Further analysis of the gene annotated as O-methyltransferase (OMT) resulted in a total of 61 sequences. KEGG pathway enrichment analysis showed that these OMT genes were enriched in the phenylpropanoid metabolism pathway. Methyleugenol is biosynthesized in the phenylpropanoid metabolism pathway, and EOMT is the last enzyme that in methyleugenol biosynthesis pathway. Therefore, these unigenes can be used as candidate genes to identify and study the function of EOMT.

### 4.3. Drought Stress Induced the Expression of Which Was Hypothetically Involved in the Biosynthesis of Safrole the Synthesis of Safrole

In plants, safrole was speculated to be produced from eugenol and catalyzed by CYP719A [16]. By screening the annotated unigene data, we found four genes annotated to CYP719A, namely CYP719A5 (c40351.graph_c1), CYP719A1 (c67694.graph_c0, c40351.graph_c0 and c40910.graph_c0). Interestingly, the expression value of the genes, encoding these two enzymes, showed an opposite changing trend. In roots, the expression levels of the gene, encoding CYP719A5, first increased and then decreased, while the expression levels of the other three genes, encoding CYP719A1, decreased early on and then increased. It was inferred that the two enzymes CYP719A5 and CYP719A1 were involved in different regulatory pathways in response to drought stress. The expression level of the gene encoding CYP719A5 (c40351.graph_c1) was consistent with the dynamic change of the content of safrole, suggesting that CYP719A5 was involved in the transformation from eugenol into safrole.

It has been reported that the heterologous expression of CYP719A5 in yeast expresses the activity of cheilanthifoline synthase, catalyzes the formation of methylenedioxy bridge from (S)-scoulerine to cheilanthifoline [52]. The structural change from eugenol to safrole was a methylene dioxane bridge on the benzene ring. The changes in the content of safrole and the expression level of CYP719A5 (c40351.graph_c1) were consistent. This result seemed to agree with the catalytic activity of the heterologous expression of CYP719A5, reported in the literature [52]. Our results predict that the toxic component safrole, in *A. sieboldii*, was synthesized from eugenol by a c40351.graph_c1 gene-encoded CYP719A5 enzyme, and CYP719A1 in *A. sieboldii* may not involve in the synthesis of safrole. Therefore, c40351.graph_c1 could be used as a candidate gene for cloning and functional verification in the future.

## 5. Conclusions

In summary, we studied the genes related to methyleugenol biosynthesis in *A. sieboldii* under drought stress. The content of methyleugenol was improved under drought stress, and several genes, such as HCT, CCR and IGS, were up-regulated and correlated well with methyleugenol content. Drought stress improves the production of methyleugenol by up-regulating genes in the phenylpropanoid biosynthesis pathway, while it decreases the toxic safrole by down-regulating CYP719A5 gene. Our study will be helpful in engineering the metabolites in *A. sieboldii* to improve its medicinal value.

## Figures and Tables

**Table 1:** Length distribution of unigenes and transcripts.

Length Range	Transcript	Unigene
300–500	38,585 (16.88%)	23,455 (43.97%)
500–1000	48,103 (21.04%)	15,156 (28.41%)
1000–2000	67,681 (29.60%)	8559 (16.04%)
2000+	74,249 (32.48%)	6174 (11.57%)
Total Number	228,618	53,344
Total Length	387,891,699	51,209,482
N50 Length	2423	1469
Mean Length	1696.68	959.99

**Table 2:** Unigene functional annotations.

Database	Annotated_Number	300 ≤ Length < 1000	Length ≥ 1000
COG	9620	4634	4986
GO	16,293	9510	6783
KEGG	10,789	5899	4890
KOG	16,792	9060	7732
Pfam	19,739	9586	10,153
Swiss-Prot	17,548	8390	9158
eggNOG	26,155	14,173	11,982
NR	27,995	15,693	12,302
All_Annotated	28,715	16,313	12,402

## Data Availability

All the relevant data are provided along with the manuscript as Supplementary Files.

## Supplementary Materials

The following are available online at https://www.mdpi.com/article/10.3390/biom11081233/s1, Table S1: Primer sequences for qRT-PCR, Table S2: Components identified in volatile oil, Table S3: DEGs in root and rhizome, Table S4: GO enrichment of DEGs in root and rhizome, Table S5: KEGG enrichment of DEGs in root and rhizome, Table S6: DEGs related to phenylpropanoid biosynthesis.
